# Supplementary material for: Trends in opioid prescribing practices in South Korea, 2009–2019: Are we safe from an opioid epidemic?
Source: PLoS One. 2021 May 12;16(5):e0250972. doi: 10.1371/journal.pone.0250972 (PMC8115784; doi:10.1371/journal.pone.0250972)
Supplement: S1 Table — (DOCX) [file pone.0250972.s001.docx]

**S1 Table. Trends in rate (per 1000 population) of all opioids prescribed in South Korea, 2009-2019.**

|  | All opioid Rx rate | | Change, % | AAPC (95% CI) | Trend1 |  | Trend2 |  |
| --- | --- | --- | --- | --- | --- | --- | --- | --- |
| Administrative Districts | 2009 | 2019 | 2009-2019 | 2009-2019 | Years | APC (95% CI) | Years | APC (95% CI) |
| South Korea | 347.5 | 531.3 | 52.9 | 4.3 (3.5-5.1) | 2009-2013 | 8.3 (6.2-10.5) | 2013-2019 | 1.8 (0.8-2.7) |
| Seoul | 263.0 | 464.0 | 76.5 | 5.7 (4.9-6.6) | 2009-2013 | 9.7 (7.4-12.1) | 2013-2019 | 3.2(2.1-4.2) |
| Busan | 341.6 | 607.2 | 77.7 | 6 (4.8-7.1) | 2009-2012 | 12.2 (7.7-16.9) | 2012-2019 | 3.4 (2.5-4.3) |
| Incheon | 245.4 | 429.4 | 75.0 | 6 (4.5-7.6) | 2009-2013 | 9.4 (5.3-13.7) | 2013-2019 | 3.8 (2.1-5.6) |
| Daegu | 431.3 | 727.2 | 68.6 | 5.1 (3.5-6.8) | 2009-2012 | 12.2 (5.9-18.9) | 2012-2019 | 2.3 (0.9-3.6) |
| Gwangju | 310.7 | 503.1 | 61.9 | 5.3 (4.1-6.4) | 2009-2013 | 9.4 (6.4-12.5) | 2013-2019 | 2.6 (1.3-3.9) |
| Daejeon | 315.0 | 484.2 | 53.7 | 4.4 (3.5-5.3) | 2009-2013 | 8.2 (5.8-10.6) | 2013-2019 | 2 (0.9-3) |
| Ulsan | 335.5 | 494.6 | 47.4 | 4.4 (3.1-5.6) | 2009-2015 | 6.6 (4.8-8.4) | 2015-2019 | 1.1 (-1.7-4) |
| Gyeonggi-do | 239.1 | 395.1 | 65.3 | 5.1 (4-6.2) | 2009-2013 | 8.5 (5.5-11.5) | 2013-2019 | 2.9 (1.7-4.2) |
| Gangwon-do | 349.4 | 482.9 | 38.2 | 3.4 (2.4-4.3) | 2009-2013 | 7 (4.4-9.6) | 2013-2019 | 1 (-0.2-2.2) |
| Chungcheongbuk-do | 469.7 | 661.8 | 40.9 | 3.9 (2.7-5.1) | 2009-2013 | 7.7 (4.5-11) | 2013-2019 | 1.4 (0-2.8) |
| Chungcheongnam-do | 541.3 | 614.4 | 13.5 | 1.2 (0.1-2.2) | 2009-2013 | 4.7 (2.1-7.4) | 2013-2019 | -1.1 (-2.4-0.1) |
| Jeollabuk-do | 562.9 | 797.7 | 41.7 | 4 (2.8-5.2) | 2009-2013 | 7.3 (4.3-10.4) | 2013-2019 | 1.8 (0.5-3.2) |
| Jeollanam-do | 626.0 | 889.8 | 42.1 | 3.7 (3-4.4) | 2009-2013 | 8.4 (6.6-10.2) | 2013-2019 | 0.7 (-0.1-1.5) |
| Gyeongsangbuk-do | 552.6 | 672.9 | 21.8 | 1.8 (1.3-2.4) | 2009-2012 | 7.8 (5.6-10) | 2012-2019 | -0.6 (-1.1--0.1) |
| Gyeongsangnam-do | 428.8 | 655.8 | 53.0 | 4.7 (3.3-6.1) | 2009-2013 | 10.1 (6.4-13.9) | 2013-2019 | 1.2 (-0.3-2.8) |
| Jeju-do | 409.7 | 566.3 | 38.2 | 3.3 (2-4.6) | 2009-2014 | 6.9 (4.4-9.5) | 2014-2019 | -0.2 (-2.2-1.8) |
| Sejong-si |  | 300.4 |  | -7 (-13.8-0.4) | 2012-2019 | -7 (-13.8-0.4) |  |  |

AAPC, average annual percent change; APC, annual percent change; Rx, prescription
