# Supplementary material for: Trends in opioid prescribing practices in South Korea, 2009–2019: Are we safe from an opioid epidemic?
Source: PLoS One. 2021 May 12;16(5):e0250972. doi: 10.1371/journal.pone.0250972 (PMC8115784; doi:10.1371/journal.pone.0250972)
Supplement: S2 Table — (DOCX) [file pone.0250972.s002.docx]

**S2 Table. Trends in rate (per 1000 population) of strong opioids prescribed in South Korea, 2009-2019.**

|  | Strong opioid Rx rate | | Change, % | AAPC (95% CI) | Trend1 |  | Trend2 |  |
| --- | --- | --- | --- | --- | --- | --- | --- | --- |
| Administrative Districts | 2009 | 2019 | 2009-2019 | 2009-2019 | Years | APC (95% CI) | Years | APC (95% CI) |
| South Korea | 0.6 | 15.2 | 2433.3 | 46.3 (32.6-61.5) | 2009-2013 | 116 (60.3-191.1) | 2013-2019 | 12.8 (7.2-18.8) |
| Seoul | 0.9 | 24.2 | 2647.7 | 49.1 (33.1-66.9) | 2009-2013 | 126.1 (60.5-218.4) | 2013-2019 | 12.9 (6.6-19.7) |
| Busan | 1.9 | 15.4 | 693.8 | 25.4 (21.2-29.8) | 2009-2013 | 53.2 (38.7-69.1) | 2013-2019 | 9.7 (6.8-12.7) |
| Incheon | 0.6 | 13.1 | 2120.3 | 34.5 (23.1-47) | 2009-2015 | 57.4 (32.8-86.5) | 2015-2019 | 6.3 (-4.8-18.7) |
| Daegu | 0.7 | 23.5 | 3307.2 | 47.1 (33.2-62.5) | 2009-2013 | 127.1 (67.8-207.4) | 2013-2019 | 10.2 (5-15.5) |
| Gwangju | 0.7 | 11.1 | 1440.3 | 31.9 (15.8-50.3) | 2009-2015 | 54 (20.5-96.9) | 2015-2019 | 4.6 (-12.2-24.6) |
| Daejeon | 0.5 | 15.5 | 3333.3 | 43.2 (27.4-61) | 2009-2015 | 66.8 (32.8-109.5) | 2015-2019 | 13.9 (0.2-29.4) |
| Ulsan | 1.2 | 15.3 | 1198.3 | 23.3 (15.4-31.7) | 2009-2016 | 33.9 (22.3-46.7) | 2016-2019 | 1.7 (-14.6-21.1) |
| Gyeonggi-do | 0.3 | 11.0 | 4115.4 | 59.1 (34.5-88.2) | 2009-2013 | 163.5 (57.4-341.1) | 2013-2019 | 13.7 (6.4-21.5) |
| Gangwon-do | 0.1 | 14.5 | 23983.3 | 96 (48.6-158.6) | 2009-2013 | 304.3 (71.1-855.3) | 2013-2019 | 21 (13.9-28.5) |
| Chungcheongbuk-do | 0.9 | 11.4 | 1163.3 | 36.7 (20.6-54.9) | 2009-2014 | 79.1 (33.8-139.7) | 2014-2019 | 4.3 (-6.9-16.8) |
| Chungcheongnam-do | 0.9 | 14.8 | 1584.1 | 39.9 (24.1-57.7) | 2009-2014 | 80.5 (36.2-139.1) | 2014-2019 | 8.4 (-2.1-20.1) |
| Jeollabuk-do | 0.8 | 24.2 | 3124.0 | 42.7 (27.7-59.4) | 2009-2015 | 64.5 (32.6-104.1) | 2015-2019 | 15.3 (1.8-30.5) |
| Jeollanam-do | 0.7 | 8.8 | 1157.1 | 38 (17.9-61.5) | 2009-2013 | 77.3 (11.3-182.5) | 2013-2019 | 16.7 (5.1-29.5) |
| Gyeongsangbuk-do | 0.2 | 10.6 | 6993.3 | 49.5 (34.8-65.7) | 2009-2014 | 124.9 (75-189) | 2014-2019 | -0.7 (-6-5) |
| Gyeongsangnam-do | 0.2 | 10.8 | 7106.7 | 49.8 (32.9-68.8) | 2009-2013 | 142.9 (68.7-249.7) | 2013-2019 | 8.5 (3-14.3) |
| Jeju-do | 0.0 | 7.2 | 72000.0 | 129.1 (27.6-311.2) | 2009-2013 | 495.6 (-4-3593.8) | 2013-2019 | 21.2 (16.3-26.2) |
| Sejong-si |  | 1.1 |  | -4.4 (-20.7-15.4) | 2012-2019 | -4.4 (-20.7-15.4) |  |  |

AAPC, average annual percent change; APC, annual percent change; Rx, prescription
