# Supplementary material for: Trends in opioid prescribing practices in South Korea, 2009–2019: Are we safe from an opioid epidemic?
Source: PLoS One. 2021 May 12;16(5):e0250972. doi: 10.1371/journal.pone.0250972 (PMC8115784; doi:10.1371/journal.pone.0250972)
Supplement: S3 Table — (DOCX) [file pone.0250972.s003.docx]

**S3 Table. Trends in rate (per 1000 population) of ER/LA opioids prescribed in South Korea, 2009-2019.**

|  | LA/ER opioid Rx, rate | | Change, % | AAPC (95% CI) | Trend1 |  | Trend2 |  |
| --- | --- | --- | --- | --- | --- | --- | --- | --- |
| Administrative Districts | 2009 | 2019 | 2009-2019 | 2009-2019 | Years | APC (95% CI) | Years | APC (95% CI) |
| South Korea | 6.8 | 47.5 | 598.5 | 31.8 (25.3-38.6) | 2009-2014 | 64.1 (46.2-84.1) | 2014-2019 | 5.9 (0.7-11.3) |
| Seoul | 6.0 | 91.2 | 1419.8 | 34.3 (27.4-41.6) | 2009-2014 | 66.4 (47.4-88) | 2014-2019 | 8.4 (2.9-14.1) |
| Busan | 9.7 | 94.2 | 873.7 | 26.2 (21.2-31.4) | 2009-2014 | 50.7 (37.6-65) | 2014-2019 | 5.7 (1.1-10.5) |
| Incheon | 4.3 | 64.8 | 1424.5 | 36.6 (28.2-45.6) | 2009-2014 | 73.8 (49.8-101.7) | 2014-2019 | 7.4 (1.5-13.7) |
| Daegu | 7.1 | 89.3 | 1153.8 | 31 (23.1-39.3) | 2009-2013 | 83.5 (52.8-120.4) | 2013-2019 | 4.6 (0.4-8.9) |
| Gwangju | 7.0 | 99.9 | 1334.8 | 31.8 (25.6-38.3) | 2009-2014 | 63.6 (46.5-82.7) | 2014-2019 | 6.2 (1.3-11.4) |
| Daejeon | 7.7 | 67.2 | 772.9 | 25.9 (21.3-30.8) | 2009-2014 | 47.9 (35.8-61) | 2014-2019 | 7.3 (2.9-11.8) |
| Ulsan | 10.7 | 55.2 | 418.4 | 23 (16.4-30) | 2009-2015 | 40.6 (28-54.6) | 2015-2019 | 0.7 (-8.8-11.1) |
| Gyeonggi-do | 3.6 | 65.0 | 1700.3 | 36.4 (29.5-43.8) | 2009-2014 | 73.2 (53.3-95.7) | 2014-2019 | 7.5 (2.6-12.5) |
| Gangwon-do | 10.0 | 93.7 | 839.3 | 31.7 (21.4-42.8) | 2009-2014 | 57.9 (31.5-89.7) | 2014-2019 | 9.7 (0.7-19.6) |
| Chungcheongbuk-do | 8.7 | 87.9 | 912.4 | 31 (23.6-38.9) | 2009-2014 | 62.6 (42.2-85.9) | 2014-2019 | 5.6 (-0.4-12) |
| Chungcheongnam-do | 6.1 | 85.5 | 1296.2 | 33.6 (25.2-42.6) | 2009-2014 | 78.1 (53.1-107.1) | 2014-2019 | 0.3 (-5.7-6.6) |
| Jeollabuk-do | 7.8 | 112.4 | 1342.6 | 35.9 (25.9-46.7) | 2009-2014 | 68.5 (41.2-101.1) | 2014-2019 | 9.6 (1.9-17.8) |
| Jeollanam-do | 14.1 | 110.5 | 682.5 | 28.7 (17.1-41.5) | 2009-2014 | 62.7 (31.7-101.2) | 2014-2019 | 1.8 (-8.4-13.2) |
| Gyeongsangbuk-do | 8.0 | 103.6 | 1193.6 | 34.8 (26.2-44) | 2009-2014 | 74.8 (50-103.7) | 2014-2019 | 4 (-2.2-10.6) |
| Gyeongsangnam-do | 10.2 | 76.6 | 652.4 | 26.1 (17.3-35.6) | 2009-2014 | 51.6 (29-78.1) | 2014-2019 | 4.9 (-3.3-13.8) |
| Jeju-do | 6.3 | 78.8 | 1149.4 | 35.1 (20.1-52.1) | 2009-2014 | 76.3 (34.2-131.6) | 2014-2019 | 3.6 (-7.4-15.9) |
| Sejong-si |  | 20.4 |  | -10.8 (-21.7-1.7) | 2012-2019 | -10.8 (-21.7-1.7) |  |  |

AAPC, average annual percent change; APC, annual percent change; Rx, prescription
