# Supplementary material for: Trends in opioid prescribing practices in South Korea, 2009–2019: Are we safe from an opioid epidemic?
Source: PLoS One. 2021 May 12;16(5):e0250972. doi: 10.1371/journal.pone.0250972 (PMC8115784; doi:10.1371/journal.pone.0250972)
Supplement: S4 Table — (DOCX) [file pone.0250972.s004.docx]

|  | **population^a^** | **total opioid prescriptions^b^** | **prescriptions_1000** | **ER/LA prescriptions^b^** | **ER/LA_1000** |
| --- | --- | --- | --- | --- | --- |
| **2006** | 298,379,912 | 215,917,091 | 723.6315 | 19,000,704 | 63.67957 |
| **2007** | 301,231,207 | 228,543,586 | 758.6982 | 21,026,010 | 69.80024 |
| **2008** | 304,093,966 | 237,860,047 | 782.1926 | 22,358,844 | 73.5261 |
| **2009** | 306,771,529 | 243,741,861 | 794.5387 | 22,667,993 | 73.8921 |
| **2010** | 309,321,666 | 251,095,243 | 811.7609 | 23,351,858 | 75.49377 |
| **2011** | 311,556,874 | 252,175,391 | 809.404 | 22,947,961 | 73.65577 |
| **2012** | 313,830,990 | 255,215,911 | 813.2272 | 22,459,000 | 71.564 |
| **2013** | 315,993,715 | 247,097,560 | 781.9699 | 22,238,780 | 70.37729 |
| **2014** | 318,301,008 | 240,993,021 | 757.123 | 21,930,365 | 68.89819 |
| **2015** | 320,635,163 | 226,819,924 | 707.4081 | 21,547,893 | 67.20377 |
| **2016** | 322,941,311 | 214,236,023 | 663.39 | 20,352,422 | 63.02205 |
| **2017** | 324,985,539 | 191,218,266 | 588.3901 | 17,400,862 | 53.5435 |

**S4 Table. The rate of prescriptions per 1000 persons in the United States, 2006-2017.**

ER/LA, extended release and long acting.

^a^ Annual estimates of the resident population for the United States, <http://census.gov>

^b^ From Schieber, L. Z., Guy, G. P., Seth, P., Young, R., Mattson, C. L., Mikosz, C. A., & Schieber, R. A. (2019). Trends and patterns of geographic variation in opioid prescribing practices by state, United States, 2006-2017. *JAMA network open*, *2*(3), e190665-e190665.
