# Supplementary material for: Trends in opioid prescribing practices in South Korea, 2009–2019: Are we safe from an opioid epidemic?
Source: PLoS One. 2021 May 12;16(5):e0250972. doi: 10.1371/journal.pone.0250972 (PMC8115784; doi:10.1371/journal.pone.0250972)
Supplement: S5 Table — (DOCX) [file pone.0250972.s005.docx]

**S5 Table. The rate of prescriptions of strong opioids per 1000 persons in the United States, 2006-2009.**

| **year** | **population** | **fentanyl ER^b^** | **fentanyl IR^b^** | **hydrocodone^b^** | **hydromorphone^b^** | **morphine ER^b^** | **morphine IR^b^** | **oxycodone ER^b^** | **oxycodone IR^b^** | **Ratio_Strong Opioids^c^** |
| --- | --- | --- | --- | --- | --- | --- | --- | --- | --- | --- |
| **2006** | 298,379,912 | 1.59 | 0.12 | 38.23 | 0.47 | 1.25 | 0.4 | 2.33 | 10.34 | 165 |
| **2007** | 301,231,207 | 1.72 | 0.11 | 40.11 | 0.54 | 1.39 | 0.43 | 2.5 | 11.52 | 182.1 |
| **2008** | 304,093,966 | 1.77 | 0.1 | 40.92 | 0.62 | 1.59 | 0.47 | 2.57 | 12.79 | 199.1 |
| **2009** | 306,771,529 | 1.64 | 0.08 | 40.1 | 0.71 | 1.68 | 0.42 | 2.52 | 13.62 | 206.7 |

ER, extended release; IR, immediate release

^a^ Annual estimates of the resident population for the United States, <http://census.gov>

^b^ Rate of prescription per 100 persons from Kenan, K., Mack, K., & Paulozzi, L. (2012). Trends in prescriptions for oxycodone and other commonly used opioids in the United States, 2000–2010. *Open Medicine*, *6* e41.

^C^ Converted to rate per 1000 persons.
