# Supplementary material for: Trends in opioid prescribing practices in South Korea, 2009–2019: Are we safe from an opioid epidemic?
Source: PLoS One. 2021 May 12;16(5):e0250972. doi: 10.1371/journal.pone.0250972 (PMC8115784; doi:10.1371/journal.pone.0250972)
Supplement: Attachment — Submitted filename: Untitled 3.pdf [file pone.0250972.s006.pdf]

63 “no issue” clarify statement. references?
71 “free from epidemic; how is this defined
91 state definition of “strong” here, explain how this relates to MME/day and limits of not using MME/day
124 “each year” - actually the mean value, clarify this. Is this meaningful?
126 “The number of total opioid prescriptions in 2019 was equal to 53% of the population of South Korea.”
128 In 2012 to 2013 there is a large change. This is the most important issue that needs to be addressed in the revision: why did this occur? Was there a change in data used? Was there a change in governmental regulation of opioids? Was there a change of opioid availability? Were new opioid products being introduced into the market? Were some drugs reclassified as opioids? Was there a change in how much could be dispensed by pharmacies, hence more prescriptions for the same amount of opioid? And so forth… The issue is that the paper rests on the tenuous data on the number of prescriptions and there is a significant change at this time. This may not correlate with increasing MME/day or with abuse, misuse, or overdose deaths. A thorough explanation of the reasons for this change must be explored to justify any conclusions drawn.
130 Was Sejong-si an outlier in terms of opioid prescription that could account for per capita changes?
255 greatest “percentage” increase
264 access must be addressed. Were pharmacies and specialist prescribers distributed equally in all districts? Did, for example, high prescription districts have major cancer treatment centers that others did not, etc.
265-269 simply incorrect. ER/LA opioids in the USA are problematic because the MME/day is much higher in these preparations. It is not known if this is the case in South Korea or if the ER/LA prescriptions written in South Korea correlate with higher MME/day prescribed. Without knowing this, no conclusion can be drawn about the need for regulation
276 “addiction to illicit opioids”
278-279 Possibly true but this conclusion cannot be drawn from the data presented. Revise “is needed” to “should be considered”
280-298 The main driving issue for regulatory change in the USA was overdose deaths - morbidity and mortality are not addressed in this data, and it is suggested that a national database of such data be established if it does not exist.
